# High-Throughput Polar Code Decoders with Information Bottleneck Quantization

**DOI:** 10.3390/e26060462

**Published:** 2024-05-28

**Authors:** Claus Kestel, Lucas Johannsen, Norbert Wehn

**Affiliations:** Microelectronic Systems Design Research Group, RPTU Kaiserslautern-Landau, 67663 Kaiserslautern, Germany; lucas.johannsen@rptu.de (L.J.); norbert.wehn@rptu.de (N.W.)

**Keywords:** forward error correction, polar code, information bottleneck, ASIC, 12 nm, implementation

## Abstract

In digital baseband processing, the forward error correction (FEC) unit belongs to the most demanding components in terms of computational complexity and power consumption. Hence, efficient implementation of FEC decoders is crucial for next-generation mobile broadband standards and an ongoing research topic. Quantization has a significant impact on the decoder area, power consumption and throughput. Thus, lower bit widths are preferred for efficient implementations but degrade the error correction capability. To address this issue, a non-uniform quantization based on the Information Bottleneck (IB) method is proposed that enables a low bit width while maintaining the essential information. Many investigations on the use of the IB method for Low-density parity-check code) LDPC decoders exist and have shown its advantages from an implementation perspective. However, for polar code decoder implementations, there exists only one publication that is not based on the state-of-the-art Fast Simplified Successive-Cancellation (Fast-SSC) decoding algorithm, and only synthesis implementation results without energy estimation are shown. In contrast, our paper presents several optimized Fast-SSC polar code decoder implementations using IB-based quantization with placement and routing results using advanced 12 nm FinFET technology. Gains of up to 16% in area and 13% in energy efficiency are achieved with IB-based quantization at a Frame Error Rate (FER) of $10^{-7}$ and a polar code of N=1024,R=0.5 compared to state-of-the-art decoders.

## 1. Introduction

Polar codes are a relatively new class of Forward Error Correction (FEC) codes, first described by Erdal Arıkan in 2009 [1]. These codes are part of the 5G standard. They offer low-complexity encoding and decoding algorithms, which is especially important for high-throughput and low-latency applications in upcoming standards such as 6G [2]. The most commonly used decoding algorithms for polar codes, Successive-Cancellation (SC) and Successive-Cancellation List (SCL), can be efficiently pipelined to achieve very high throughput and low latency [3,4]. Quantization has a significant impact on implementation costs. Coarse quantization improves implementation efficiency in terms of area, power and throughput but may decrease the error correction performance. Finding a good trade-off is therefore essential for efficient hardware implementations.

One promising technique to maintain the message information but enable a reduction in the bit width is the Information Bottleneck (IB) method [5]. Here, information compression is achieved by maximizing the mutual information between an observed and a compressed random variable for a given bit width. This yields a non-uniform quantization. While IB-based quantization for Low Density Parity Check (LDPC) decoder implementations is well investigated [6,7,8], the efficiency of the IB method for polar code decoders is quite unexplored. It is an open research question whether IB-based quantization in polar decoders can yield more efficient implementations compared to standard quantization methods.

It was shown in [8,9] that a pure lookup table (LUT)-based application of the IB method yields a very large LUTs, making this approach unfeasible. The Reconstruction Computation Quantization (RCQ) scheme [10] also uses a LUTs to reconstruct and, after computation, quantize back to a smaller bit width. The resulting LUTs have much lower complexity. Hence, the RCQ scheme is the most promising approach from an implementation perspective. To the best of our knowledge, only one publication has investigated the efficiency of IB-based polar decoder implementation [9]. However, the investigations in [9] do not consider the state-of-the-art Fast-SSC decoding algorithm and, even more importantly, provide only synthesis results without any power data, which are some of the most important implementation metrics.

This work therefore makes the following new contributions:

- We present the first Fast Simplified Successive Cancellation (Fast-SSC) polar decoder architecture using IB-based quantization with optimized LUTs to improve area and energy efficiency;
- We compare the non-uniform, IB-based quantization scheme with uniformly quantized fixed-point (FP) representations in terms of error correction performance and implementation efficiency for code lengths of 128 bit and 1024 bit;
- We analyze the impact of the IB-based quantization on area and power consumption with seven different designs in advanced 12 nm FinFET technology.

The remainder of this paper is structured as follows: We provide the required background of polar codes, their decoding algorithms and the IB method in Section 2. IB-based Fast-SSC decoding and our decoder architecture are described in Section 3. Section 4 presents a detailed comparison with uniformly quantized FP decoders in terms of error correction performance and implementation costs, and Section 5 concludes this paper.

## 2. Background

### 2.1. Polar Codes

Polar codes P(N,K) are linear block codes with code length $N=2^{n}$ that encode *K* information bits. Channel polarization derives *N* virtual channels where *K* reliable channels (information set I) are used to transmit the information. The N−K remaining (unreliable) channels are set to zero and called frozen bits (frozen set F). The encoding includes a bit reversal permutation [1].

### 2.2. Successive Cancellation Decoding

SC decoding can be described as depth-first tree traversal of the Polar Factor Tree (PFT) [11]. The PFT has log(N)+1 stages *s* and *N* leaf nodes at stage s=0, representing the frozen and information bits. Each node *v* receives an Log-Likelihood Ratio (LLR) vector $\alpha^{v}$ of size $N_{s}$ to first calculate the $N_{s}/2$ elements of the left child message $\alpha^{l}$ using the hardware-efficient min-sum formulation of the *f*-function:

(1) $$\begin{array}{ccc} \alpha_{i}^{l} & =f(\alpha_{2i}^{v},\alpha_{2i+1}^{v}) & =\mathrm{sign}\left(\alpha_{2i}^{v}\right)\mathrm{sign}\left(\alpha_{2i+1}^{v}\right)\min\left(\left|\alpha_{2i}^{v}\right|,\left|\alpha_{2i+1}^{v}\right|\right). \end{array}$$

With the bit vector $\beta^{l}$ received from the left child, the $N_{s}/2$ elements of $\alpha^{r}$ are calculated using the *g*-function and sent to the child on the right:

(2) $$\alpha_{i}^{r}=g\left(\alpha_{2i}^{v},\alpha_{2i+1}^{v},\beta_{i}^{l}\right)=\left(1-2\beta_{i}^{l}\right)\;\cdot \;\alpha_{2i}^{v}+\alpha_{2i+1}^{v},$$

With the results of both children, the *h*-function calculates the partial sum $\beta^{v}$ with ⊕, the binary XOR operator, as

(3) $$\left(\beta_{2i}^{v},\beta_{2i+1}^{v}\right)=h\left(\beta_{i}^{l},\beta_{i}^{r}\right)=\left(\beta_{i}^{l}⊕\beta_{i}^{r},\beta_{i}^{r}\right),$$

which is sent to the parent node. In leaf nodes, bit decisions are made. Frozen bits are 0 per definition, and information bit nodes return the Hard Decision (HD) on the LLR:

(4) $$\beta^{v}=\mathrm{sign}\left(\alpha^{v}\right)≜\left\{\begin{array}{cc} 0 & \mathrm{if}\;\alpha^{v}\geq 0\\ 1 & \mathrm{otherwise}. \end{array}\right.$$

The decoder outputs the partial sum $\beta^{0}$ of the root node.

### 2.3. Fast-SSC Decoding

Pruning the PFT reduces the number of operations required to decode one code word [11]. Subtrees containing only frozen bits do not have to be traversed because their decoding result is known to be an all-zero vector in advance. Such Rate-0 nodes are always left children and are merged into their parent nodes. Here, the *g*- and *h*-functions are executed with the known all-zero $\beta^{l}$, denoted by $g_{0}$ and $h_{0}$, respectively. Similarly, subtrees without any frozen bits can be decoded directly by the HD because no parity information is contained. Merging these Rate-1 nodes results in the $h_{1}$-function, which directly calculates $\beta^{r}$ using

(5) $$\beta_{i}^{r}=\mathrm{sign}\left(g\left(\alpha_{2i}^{v},\alpha_{2i+1}^{v},\beta_{i}^{l}\right)\right),$$

and combines it with $\beta^{l}$ to observe $\beta^{v}$.

Fast-SSC decoding [12] applies further optimizations: If a subtree contains only one information bit, it is considered an Repetition (REP) code and replaced by a specialized REP node. All its bits are decoded by summing up the vector $\alpha^{v}$ of received LLR values and extracting the sign bit of the sum:

(6) $$\beta_{i}^{v,\mathrm{REP}}=\mathrm{sign}\left(\sum_{j=0}^{N_{s}-1}\alpha_{j}^{v}\right).$$

In subtrees containing only one frozen bit, this bit always acts as a parity bit. Thus, the partial sum of this subtree represents an Single Parity-Check (SPC) code. A specialized SPC node performs Maximum Likelihood (ML) decoding by calculating the parity $\gamma^{v}\in \left\{0,1\right\}$ of the input:

(7) $$\gamma^{v}=\overset{N_{s}-1}{\underset{i=0}{⨁}}\mathrm{sign}\left(\alpha_{i}^{v}\right),$$

finding the least reliable bit

(8) $$i_{\min}=\underset{i\in [0,N_{s})}{arg min}\left|\alpha_{i}^{v}\right|$$

and setting $\beta^{v}$ to satisfy the single parity constraint

(9) $$\beta_{i}^{v,\mathrm{SPC}}=\left\{\begin{array}{cc} \mathrm{sign}\alpha_{i}^{v}⊕\gamma^{v} & \mathrm{if}\;i=i_{\min}\\ \mathrm{sign}\alpha_{i}^{v} & \mathrm{otherwise}. \end{array}\right.$$

### 2.4. Information Bottleneck Method

The IB method is a mathematical framework used for clustering in information theory and machine learning [5]. In the IB setup, the target is to preserve the shared mutual information I(X;Y) between an observed random variable *Y* and the relevant random variable *X* while compressing *Y* to *T*, i.e., maximizing I(T;X). Different IB algorithms exist [13] to provide the compression mapping p(t|y) derived only from the joint distribution p(x;y) and the cardinality of the compressed event space (|T|), with x∈X={0,1},y∈Y,t∈T being realizations of the random variables X,Y,T, respectively, and |T|≪|Y|. A collection of IB algorithms is provided by [14] and is used in this work. This compression is applied to the output of an Additive White Gaussian Noise (AWGN) channel to obtain a coarse, non-uniform quantization.

In the case of our IB decoder, y∈Y are the received LLRs that are quantized with a high bit width, e.g., 10 bit, which equals $\left|Y\right|=2^{10}=1024$ bins. The IB algorithm then iteratively tries to find pairs of bins to combine into one bin with the least loss of mutual information I(X;Y). In that way, |Y| is reduced to |T|=16 (4 bit), and a mapping from *y* to *t* is derived.

## 3. Information Bottleneck Decoder

### 3.1. Numerical Representations and Lookup Table Generation

In this paper, we focus on decoders with very high throughput and low latency. These decoders are fully unrolled and pipelined and use an SignMagnitude (SM) representation of the quantized FP LLR values [15]. For a high Signal-to-Noise-Ratios (SNRs), saturation reduces signal toggling because only the sign bit changes, which reduces power consumption. Additionally, the comparators in the *f*-functions (1) and SPC nodes (8) can directly operate on the magnitude. To perform the additions in the *g*-functions (2) and REP nodes (6), the SM representations are converted to Two’s-Complement (TC) to efficiently perform the calculations.

Our IB decoder implementations exploit all these optimizations. However, because of the non-uniform distribution characterizing the IB indices, mathematical computations must be replaced by LUTs [9]. These LUTs can become extremely large. For example, for a *g*-function (2) with one binary and two LLR inputs, the resulting LUT is of size ${2\;\cdot \;|T|}^{2}$. From an implementation perspective, the LUTs are transformed into Boolean functions. Despite the logic optimization executed by state-of-the-art synthesis tools, the resulting logic costs can quickly outweigh the benefits of reduced bit widths, particularly for increasing LUT sizes [8].

A promising approach to address this problem is the RCQ scheme [10,16]. RCQ combines non-uniform quantization with the traditional node computations. Only small LUTs are necessary and are placed in front of the computation units to upscale the reduced non-uniform IBquantization to a uniform FP quantization (*Reconstruction*). Node computations are then performed with the uniform FP quantization (*Computation*). After computation, the results are downscaled back to the non-uniform IB domain with smaller bit width (*Quantization*). The RCQ scheme results in much smaller LUTs because the mappings for the conversions between IB and FP domains can be implemented as LUTs of size $2^{Q}$ for each value where *Q* is the bit width. We use $Q_{\mathrm{IB}}=\log_{2}\left(|T|\right)$ and $Q_{\mathrm{FP}}$ to denote the bit widths in the IB and FP domains, respectively. For the *g*-function example mentioned above, the number of entries in the LUTs shrinks from ${2\;\cdot \;|T|}^{2}=2^{1+2\;\cdot \;Q_{\mathrm{IB}}}$ to $2\;\cdot \;2^{Q_{\mathrm{IB}}}+2^{Q_{\mathrm{FP}}}$.

**Figure 1:**
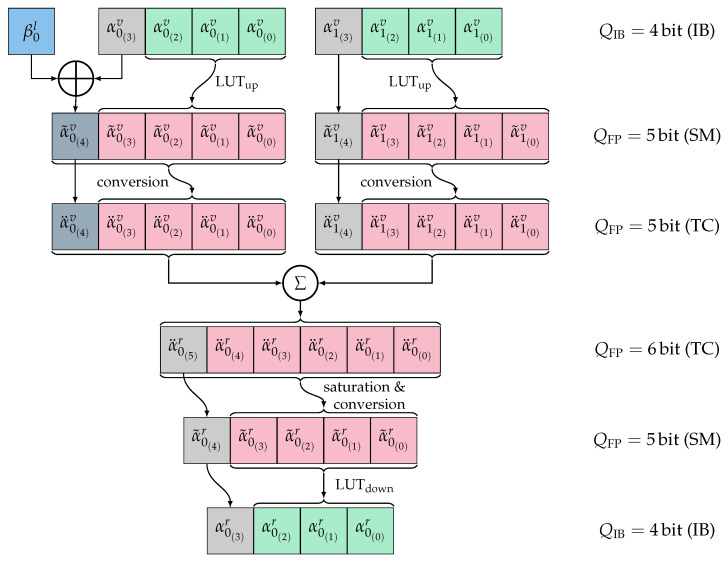
RCQ schematic for the g-function.

We use density evolution to generate samples. At least 100K samples are monitored at each node in the decoder, i.e., at each edge of the PFT. These samples yield the joint distributions p(x;y), which are input into the IBalgorithm [13] that calculates the compression mappings p(t|y) for every edge. Then, the *symmetric information bottleneck algorithm* [7,14] is applied, which is optimized to preserve the symmetry of the transmission channel (assuming an AWGN channel and Binary Phase Shift Keying (BPSK) modulation). Exploiting this symmetry enables a bisection of the LUTs because it is sufficient to store only the magnitudes. Accordingly, we use an SM-like representation of the IB indices, as shown in the example in Table 1, for |T|=8. Therefore, for both directions of conversion (IB to FP and FP to IB), the LUTs directly map one magnitude to another, i.e., the magnitudes in both domains also act as indices for the LUTs. Thus, the size of each LUT is $2^{Q-1}$, and the total number of entries for the example of the *g*-function becomes $2\;\cdot \;2^{Q_{\mathrm{IB}}-1}+2^{Q_{\mathrm{FP}}-1}$. As shown in Figure 1, this means a reduction from $2*16^{2}=512$ entries to $2*2^{4-1}$ for the upscaling LUT and $2^{5-1}$ for the downscaling LUT, making it just 32 entries.

Furthermore, this approach eliminates the need for multiple comparisons with thresholds per conversion as in [16].

#### Notation:

To differentiate between the numerical representations, we use α to denote values in the IB domain and $\tilde{\alpha }$ and $\ddot{\alpha }$ for SM and TC FP representations, respectively. The *j*-th bit of the binary expansion of $\alpha_{i}$ is given by $\alpha_{i_{\left(j\right)}}$, and the most significant bit (MSB) Q−1 refers to the sign bit.

### 3.2. Information-Bottleneck-Based Fast-SSC Decoding

#### 3.2.1. *f*-Function

With the symmetric mapping and inherent order of the IB indices (Table 1), the *f*-function (1) can be directly performed in the non-uniform IB domain, and no LUTs are necessary, which corresponds to the “*re-MS-IB* decoder” implementation of [9]. In contrast to [9], we map the IB indices so that negative values also correspond to a negative LLRs and, thus, do not need to invert the result of the *XOR*ed sign bits.

#### 3.2.2. *g*-Function

As in [16], we apply an RCQ scheme, but based on our optimized up- and downscale LUTs:

(10) $$\alpha_{i}^{r}={\mathrm{LUT}}_{\mathrm{down}}^{v}\left(g\left({\mathrm{LUT}}_{\mathrm{up}}^{v}\left(\alpha_{2i}^{v}\right),{\mathrm{LUT}}_{\mathrm{up}}^{v}\left(\alpha_{2i+1}^{v}\right),\beta_{i}^{l}\right)\right).$$

The internal separation between the different number representations is maintained for the reasons described in Section 3.1 and shown in Figure 1. The *Reconstruction* with ${\mathrm{LUT}}_{\mathrm{up}}^{v}$ maps the magnitude of the IB index $\alpha_{i}^{v}$ to its SM representation ${\tilde{\alpha }}_{i}^{v}$ with preserved sign bit. $\beta_{i}^{l}$ must be considered before conversion to the TC representation ${\ddot{\alpha }}_{i}^{v}$ for the *Computation* step. The result ${\ddot{\alpha }}_{i}^{r}$ has a 1 bit-larger resolution, implying a saturation for the conversion back to an SM representation with $Q_{\mathrm{FP}}$ bit. The *Quantization* step is again realized as magnitude ${\mathrm{LUT}}_{\mathrm{down}}^{v}$ for the transformation back to the IB domain. For the special case of merged Rate-0 nodes, i.e., the $g_{0}$-function, the *XOR*-operation with $\beta_{i}^{l}$ is omitted.

#### 3.2.3. Repetition Nodes

REP nodes calculate the sum over all input values to observe the single (repeated) information bit by HD on the sum (6). Thus, the RCQ scheme as described for the *g*-function is applied for REP nodes. An adder tree of $N_{s}-1$ adders and a depth of $s=\log_{2}N_{s}$ operates on the TC representations of the $N_{s}$ input values. The final HD as the *Quantization* step extracts the single sign bit as the bit decision of the node, for which reason no further conversion of the sum is needed.

#### 3.2.4. Single Parity Check Nodes

As in the f-function, due to the ordered mapping of the IB indices, the minimum search of the SPC node (8) can be performed directly in the IB domain. Furthermore, the chosen mapping is also suitable for the direct parity calculation (7) and the bit estimations (9) because the sign bits are preserved in the IB domain.

#### 3.2.5. $h_{1}$-Function

The $h_{1}$-function internally uses the *g*-function to compute $\beta^{r}$ (5). However, in this *g*-function, the backward conversion is not needed because the HD is made directly in the compute domain with TC representation, as already described for the REP nodes.

### 3.3. Decoder Architecture

**Figure 2:**
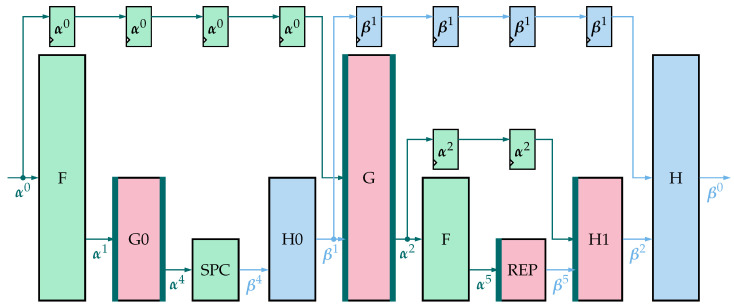
Unrolled and pipelined Fast-SSC decoder architecture for a P(16,8). Colors represent numerical domains: green for IB, red for FP and blue for binary, and dark green shows the LUTs.

An outline of the fully unrolled and deeply pipelined Fast-Simplified SC (SSC) decoder architecture for a P(16,8) is shown in Figure 2. We omit the clock signals, and the delay lines are represented by shift registers. The pipeline consists of various building blocks and implements the decoding functions described in the previous section. The IB, FP and binary domains are represented by the coloring of the blocks and signals. The decoders presented in this paper are based on the framework presented in [15], which we extended to apply the IB method as described above.

## 4. Results

We present seven decoder designs for N=128 and N=1024, which are optimized for a target frequency of 500 MHz and 750 MHz, respectively. Throughput is considered as coded throughput. The designs were synthesized with *Design Compiler* and placed and routed with *IC-Compiler*, both from *Synopsys*, in 12 nm FinFET technology with a super-low $V_{t}$ transistor library from *Global Foundries* under worst-case Process, Voltage and Temperature (PVT) conditions (125 ${}^{{}^{\circ}}C$, 0.72 V) for timing and nominal case PVT (25 ${}^{{}^{\circ}}C$, 0.8 V) for power. Error correction performance was simulated for an AWGN channel and BPSK modulation with a minimum of 100 erroneous code words.

### 4.1. Decoder for the P(128,64) Code

**Figure 3:**
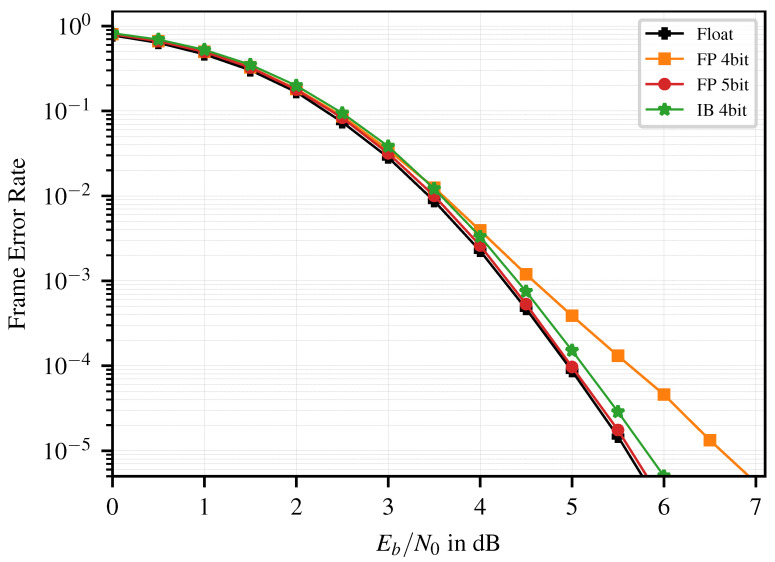
FER of the P(128,64), float vs. FP vs. IB quantization.

The Frame Error Rate (FER) of the P(128,64) is shown in Figure 3. The IB decoder with 4 bit and the FP decoder with 5 bit show similar error correction performance, whereas the error correction performance of the 4 bit FP decoder starts to degrade at an FER of $10^{-3}$.

Table 2 presents the corresponding implementation results. Comparing the decoders with similar FER (IB vs. 5 bit FP), we observe a similar combinatorial area (logic), whereas the area for the memory (registers) is reduced by ~16%. This improves the area efficiency by ~7% and yields power savings and energy efficiency improvements of ~13%.

When comparing the IB decoder with the 4 bit FP decoder, the cost of the LUTs can be directly observed in the combinatorial area (0.005 mm${}^{2}$ vs. 0.003 mm${}^{2}$), while the area for the registers is identical. This cost can be considered as the price for the improved error correction performance of the IBdecoder.

As already mentioned, there exits only one other publication that gives implementation results for IB-based decoders. Since the authors of [9] only provide synthesis results for older 28 nm technology, a fair comparison is difficult. To enable at least some comparison, we scaled the results of [9] to 12 nm according to the equations of [17]. The scaled results are included in Table 2. We limited the maximum frequency to 1000 MHz, which is more reasonable since 3681 MHz (and even 1510 MHz in the original publication) is not realistic for a design in 28 nm after placement. The reasons are, first, the power consumption and power density become infeasible with this high frequency. Second, 3681 MHz is unfeasible for standard placement and routing in semi-custom design flows in 28 nm technology. Even with the scaled estimate without frequency limitation, we observed that our optimized decoders outperform [9] in throughput, latency, area and area efficiency.

### 4.2. Decoder for the P(1024,512) Code

**Figure 4:**
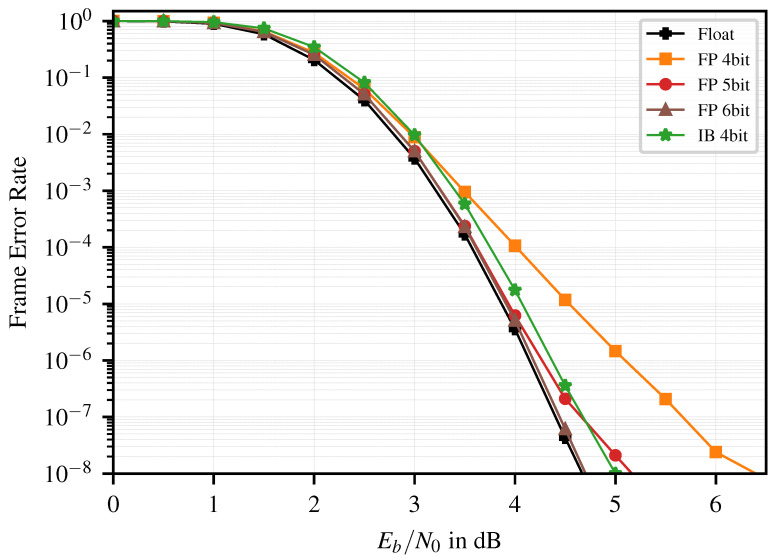
FER of the P(1024,512), float vs. FP vs. IB.

The P(1024,512) polar code has a longer pipeline and is therefore more affected by accumulating quantization errors. In contrast to the shorter code, 6 bit FP is necessary to match the floating point precision. We compare the 4 bit IB decoder to the FP decoder with the 5 bit as they show similar error correction performance (Figure 4). Here, the IB decoder even outperforms the 5 bit FP decoder at an FER of $10^{-7}$.

**Figure 5:**
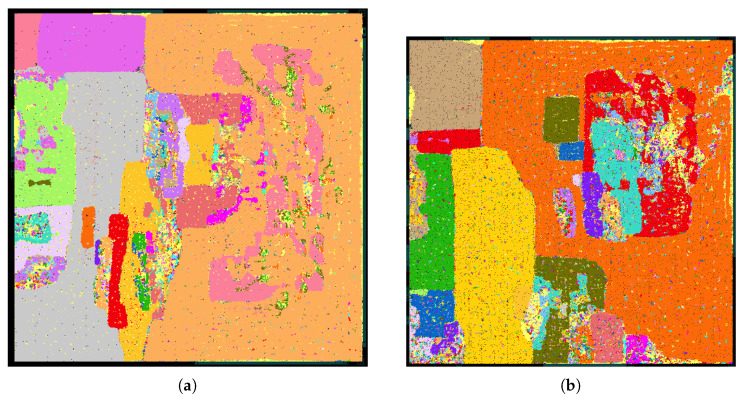
Layout pictures for FP and IB polar code decoders, same scale. (**a**) FP 5 bit: 0.968 mm${}^{2}$. (**b**) IB 4 bit: 0.822 mm${}^{2}$.

Comparing the implementation results of the IB decoder and the 5 bit FP decoder (Table 3), the total area is reduced by ~15%, mostly stemming from the reduction in registers. This leads to an improved area efficiency of ~18%, whereas the energy efficiency improves by ~15%. Figure 5 shows the layouts of the 5 bit FP and the 4 bit IB decoder.

It is also worth noting that, when compared to the close-to-float 6 bit FP decoder, accepting a small loss of ~0.2 dB in the error correction leads to improvements of ~41% in area efficiency and ~31% energy efficiency.

## 5. Conclusions

We presented fully characterized Fast-SSC polar decoders with an optimized IB-based quantization scheme. Especially for ultra-high throughput, we outperformed decoders with comparable bit width by 18% in area efficiency and 15% in energy efficiency. This effect can be mainly explained by the savings in memory requirements of fully pipelined and unrolled decoders, which was minimized with the IB-based quantization.

## Figures and Tables

**Table 1:** Hardware-aware representation of 3 bit IB indices.

IB Index *t*	0	1	2	3	4	5	6	7
SM IB	−3	−2	−1	−0	0	1	2	3
Binary	1 11	1 10	1 01	1 00	0 00	0 01	0 10	0 11

**Table 2:** Implementation results for P(128,64) decoders.

	IB 4 bit	[9] *	FP 4 bit	FP 5 bit
	Place and Route	Synthesis	Place and Route	Place and Route
Technology	12 nm	28 nm→12 nm	12 nm	12 nm
Frequency [MHz]	500	1000 (3681)	500	500
Throughput [Gbps]	64	13 (47)	64	64
Latency [ns]	18	86 (23)	18	18
Latency [CC]	9	86	9	9
Area [mm${}^{2}$]	0.014	0.026	0.012	0.015
- Registers	0.005	—	0.005	0.006
- Combinatorial	0.005	—	0.003	0.005
**Area Eff. [Gbps/mm${}^{2}$]**	**4528**	**502 (1847)**	**5384**	**4248**
Power Total [mW]	21	—	17	24
- Clock	7	—	7	9
- Registers	3	—	3	4
- Combinatorial	10	—	7	11
**Energy Eff. [pJ/bit]**	**0.32**	—	**0.27**	**0.37**
Power Density [W/mm${}^{2}$]	1.46	—	1.47	1.58

* Synthesis only, scaled from 28 nm to 12 nm (numbers in brackets) with equations from [17] and maximum frequency limited to 1000 MHz. For 28 nm and 12 nm, the scaling factors of 32 nm and 14 nm were used since they belong to the same technology generation and give the best approximation.

**Table 3:** Implementation results for P(1024,512) decoders.

	IB 4 bit	FP 4 bit	FP 5 bit	FP 6 bit
Frequency [MHz]	750	750	750	750
Throughput [Gbps]	768	768	768	768
Latency [ns]	123	123	123	123
Latency [CC]	92	92	92	92
Area [mm${}^{2}$]	0.822	0.785	0.968	1.158
- Registers	0.360	0.360	0.439	0.517
- Combinatorial	0.200	0.168	0.208	0.274
**Area Eff. [Gbps/mm${}^{2}$]**	**935**	**979**	**794**	**663**
Power Total [mW]	984	866	1149	1431
- Clock	208	207	254	298
- Registers	280	263	354	404
- Combinatorial	479	381	523	706
**Energy Eff. [pJ/bit]**	**1.28**	**1.13**	**1.50**	**1.86**
Power Density [W/mm${}^{2}$]	1.20	1.10	1.19	1.24

## Data Availability

The original contributions presented in the study are included in the article, further inquiries can be directed to the corresponding author/s.

## Abbreviations

The following abbreviations are used in this manuscript:

AWGN	Additive White Gaussian Noise
BPSK	Binary Phase Shift Keying
Fast-SSC	Fast Simplified Successive Cancellation
FEC	Forward Error Correction
FER	Frame Error Rate
FP	Fixed Point
HD	Hard Decision
IB	Information Bottleneck
LDPC	Low-Density Parity Check
LLR	Log-Likelihood Ratio
LUT	Lookup Table
ML	Maximum Likelihood
MSB	Most Significant Bit
PFT	Polar Factor Tree
PVT	Process, Voltage and Temperature
RCQ	Reconstruction Computation Quantization
REP	Repetition
SC	Successive Cancellation
SCL	Successive Cancellation List
SM	Sign Magnitude
SNR	Signal-to-Noise Ratio
SPC	Single Parity Check
SSC	Simplified SC
TC	Two’s Complement
